# Robust and Fast Whole Brain Mapping of the RF Transmit Field B1 at 7T

**DOI:** 10.1371/journal.pone.0032379

**Published:** 2012-03-12

**Authors:** Antoine Lutti, Joerg Stadler, Oliver Josephs, Christian Windischberger, Oliver Speck, Johannes Bernarding, Chloe Hutton, Nikolaus Weiskopf

**Affiliations:** 1 Wellcome Trust Centre for Neuroimaging, UCL Institute of Neurology, University College London, London, United Kingdom; 2 Special Lab Non-Invasive Brain Imaging, Leibniz Institute for Neurobiology, Magdeburg, Germany; 3 MR Centre of Excellence, Centre for Medical Physics and Biomedical Engineering, Medical University of Vienna, Vienna, Austria; 4 Otto-von-Guericke-University Magdeburg, Department Biomedical Magnetic Resonance, Institute for Experimental Physics, Magdeburg, Germany; 5 Institute for Biometry and Medical Informatics, Faculty of Medicine, Otto-von-Guericke-University, Magdeburg, Germany; University of Maryland, College Park, United States of America

## Abstract

In-vivo whole brain mapping of the radio frequency transmit field B_1_^+^ is a key aspect of recent method developments in ultra high field MRI. We present an optimized method for fast and robust in-vivo whole-brain B_1_^+^ mapping at 7T. The method is based on the acquisition of stimulated and spin echo 3D EPI images and was originally developed at 3T. We further optimized the method for use at 7T. Our optimization significantly improved the robustness of the method against large B_1_^+^ deviations and off-resonance effects present at 7T. The mean accuracy and precision of the optimized method across the brain was high with a bias less than 2.6 percent unit (p.u.) and random error less than 0.7 p.u. respectively.

## Introduction

Ultra high field (UHF) MRI has attracted an increasing level of attention over the recent years and offers interesting prospects for the future of MRI [1]. The strong inhomogeneities of the transmit RF field B_1_^+^ present in the human head at UHF lead to severe signal and contrast nonuniformities. Multi-channel transmit methods alleviate this problem and are gradually being introduced on commercial scanners thanks to the remarkable developments that have taken place in the recent years [2]–[4]. They require precise knowledge of the B_1_^+^ field to achieve homogeneous excitation and comply with safety limits [5], [6]. Quantitative mapping methods give powerful insights into biological processes. However, B_1_^+^ inhomogeneities affect most quantitative methods [7]–[9] and an accurate measure of the B_1_^+^ distributions is required for appropriate correction [10]. Robust whole-brain B_1_^+^ mapping is therefore critical for parallel transmit and quantitative mapping methods. A number of B_1_^+^ mapping methods have been introduced at field strengths ≤3T but the robustness of these methods at UHF has not been demonstrated in-vivo [11]–[16]. In this study, we present improvements to an existing 3D EPI method that yield accurate and precise whole-brain maps of the magnitude of the B_1_^+^ field at 7T. If the phase of the B_1_^+^ field is also required, the present method can be combined with an existing phase mapping technique [17]. This B_1_^+^ method was originally introduced by Jiru and Klose [15] and later optimized by Lutti et al. [16] to yield highly accurate and reproducible B_1_^+^ maps at 3T in a short acquisition time (<5 min). Although EPI readouts lead to image distortions that require offline post-processing, it is particularly suitable for 7T applications since the nominal values of the RF pulses used with this method can be set to match the large range of B_1_^+^ deviations present at 7T while the long repetition time reduces SAR levels. The improvements required for robust whole brain B_1_^+^ mapping at 7T are the increase of the dynamic range of the technique combined with parallel imaging for rapid image acquisition and the reduction of sensitivity to off-resonance effects. We assess the effect of the improvements on the accuracy and reproducibility of the B_1_^+^ maps. Using the optimal configuration we present whole-brain B_1_^+^ maps acquired in-vivo that exhibit a high level of accuracy and precision.

## Methods

### Theory

The presented method calculates distributions of B_1_^+^ fields (expressed as the local flip angle α_local_) from the ratio of stimulated echo (STE, nominal flip angle α/2) and spin echo (SE, nominal flip angle α) images acquired successively following spin excitation [15]:
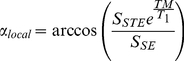
(1)where *S_SE_* and *S_STE_* are the intensities of the SE and STE images and *TM* is the time interval between the spin and stimulated echo RF pulses (*mixing time*). EPI phase images are affected by susceptibility effects and only magnitude images are used to calculate the local B_1_^+^ field. As a result, two possible values of α_local_ (90+δ and 90−δ) exist at each voxel that obey equation 1. In order to overcome this ambiguity, *N* pairs of SE and STE images are acquired with different nominal flip angle values α and the correct α_local_ values are identified as those yielding a constant α_local_/α ratio across the repetitions [15]. Here, the local B_1_^+^ values are calculated as a percentage of the nominal flip angle (in percent units = p.u.) using the hard pulse approximation [18]:
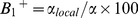
(2)As a result of the acquisition of *N* pairs of SE and STE images, *N* estimates of B_1_^+^ are calculated at each voxel. In the following, the average of these *N* values is used as a measure of the local B_1_^+^ value. The standard deviation SD_B1+_ of B_1_^+^ across the *N* repetitions is used as a measure of the uncertainty in the B_1_^+^ calculation. Note that in the experiments described below, a large number of SE/STE pairs were acquired while the number of SE/STE pairs used for calculation of the B_1_^+^ field was kept smaller.

#### Dynamic range optimization

Because S_SE_ is the denominator of equation 1, robust B_1_^+^ calculation requires maximum signal in the SE images which can be achieved by setting α to high (low) values where the local B_1_^+^ field is low (high). In a previous implementation of the method at 3T, 5 nominal values were used between 160° and 200° [16]. Due to the larger B_1_^+^ deviations present in the human head at 7T [19], whole-brain B_1_^+^ mapping requires increase of the dynamic range of the method by acquisition of data over a wider and densely sampled range of α values. In order to exclude data with low SE signal level, a sub-set of SE/STE image pairs is selected at each voxel corresponding to α values yielding maximum signal amplitude in the SE image. This voxel-specific sub-set is used for calculation of the local B_1_^+^ field.

#### Minimizing off-resonance effects

Large susceptibility-induced inhomogeneities in the polarising field B_0_ are present in the human head at 7T. The resulting off-resonance effects during spin excitation might induce significant bias in the B_1_^+^ maps due to tilting of the axis of precession and frequency dispersion effects [20]. However, these effects can be reduced by RF pulses with large amplitude and bandwidth. In previous implementations of the method, the amplitude of the RF pulses was proportional to the α values and their duration was kept constant [15], [16]. Here, we present an alternative approach (*off-resonance minimization*) where the maximally achievable RF pulse amplitude is used for all nominal values and the RF pulse duration is proportional to the nominal flip angle value. The effect of off-resonance minimization on the measured B_1_^+^ maps is demonstrated both in-vivo and using numerical simulations of the Bloch equations of off-resonance spin precession during the application of the RF pulses.

### Acquisition: general considerations

Three volunteer subjects were scanned after giving written consent according to the declaration of Helsinki. The study was approved by the ethics committee of the University of Magdeburg. 3D EPI data were acquired on a 7T whole-body system (Siemens Healthcare, Erlangen, Germany), operated with head-only CP transmit and 24-channel receive coils (Nova Medical, Inc., Wilmington MA). One B_1_^+^ map was also acquired on each subject using the Actual Flip Imaging (AFI) B_1_^+^ mapping method [12], [16]. Additional phantom experiments were conducted on another 7T whole-body system (Siemens Healthcare, Erlangen, Germany) operated with a 32-channel receive coil (Siemens Healthcare, Erlangen, Germany). Image processing was performed offline using Matlab (The MathWorks Inc., Natick, MA) version 7 and SPM8 (www.fil.ion.ucl.ac.uk).

### 3D EPI method

#### Subject acquisitions

The following parameters were used for data acquisition with the 3D EPI method: matrix size 48×64×48, image resolution 4×4×4 mm^3^, image orientation: (phase, read, partition) = (R-L, A-P, H-F). Parallel imaging (acceleration factor 2) was used along the phase and partition directions with the scanner manufacturer's GRAPPA reconstruction algorithm [21]. A fully encoded set of reference images (no undersampling) was acquired prior to the image volumes. One partition segment was sampled per readout, the echo spacing was 500 µs and the bandwidth was 2298 Hz/pixel. The echo times were 35.9 ms and 67.55 ms for the SE and STE images respectively and TM was set to 34.08 ms. For all B_1_^+^ map acquisitions, the number of α values was set to 15 (i.e. 15 SE/STE image pairs). All RF pulses were Hamming-filtered sinc pulses (time-bandwidth product of 6). A slab-selective pulse was used for spin excitation, and the SE and STE pulses were non-selective. The nominal value of the excitation RF pulse was set to α, the nominal value of the STE pulse variable across repetitions in order to maximize SNR over the entire brain. The α values were played out in a decreasing order for a fast approach to the steady state [22]. In preliminary experiments, dummy repetitions at each change of α value did not yield any visible change in the B_1_^+^ maps, suggesting rapid transition to the steady state for each new value of α. Dummy repetitions were therefore omitted in the actual experiments. An additional dataset was acquired on each subject for whole brain mapping of the B_0_ field (TE = 5 ms and 6.02 ms, TR = 667 ms, matrix size: 64×64×64, image resolution 3 mm^3^, 2 min acquisition time). The EPI image distortions were corrected using the acquired B_0_ mapping data and the toolbox described in [23]. This toolbox has been used in a large number of fMRI studies to correct for distortions of EPI time-series and was recently successfully used at 7T [24]. Distortion correction was followed by padding and smoothing of the B_1_^+^ maps, as described in [16]. The threshold for RMS padding was defined as the acceptable level of error in the B_1_^+^ calculations and was set to 5 p.u., unchanged from a previous implementation of the method at 3T [16]. Following the results of the numerical Bloch Equation simulations presented in the Results section, the threshold for B_0_ padding was set to 150 Hz (110 Hz at 3T [16]). Despite the overlap between the regions affected by the RMS and B_0_ padding, B_0_ padding was still considered necessary in order to achieve systematic correction of bias due to off-resonance precession.

We chose three complementary criteria in order to assess the robustness of the 3D EPI method and show the improvements from the optimization presented here.

A measure of the reproducibility of the 3D EPI method was obtained by calculating the voxel-wise standard deviation of the B_1_^+^ maps over three successive acquisitions.

Assessing the accuracy of the 3D EPI method requires a comparison of the B_1_^+^ maps with a gold-standard reference technique. For phantom acquisitions the long TR required by the reference acquisitions can be accommodated and such a comparison was implemented using the 2D DAM method described in (16) (see below). Due to the lack of a reference in-vivo B_1_^+^ mapping method comprehensively validated at 7T, in-vivo 3D EPI B_1_^+^ maps were compared to those obtained using the AFI method [12]. The comparison was restricted to the superior brain areas, since the limited dynamic range of the AFI method was expected to result in underperformance of the method in basal brain regions where the B_1_^+^ field is low [25].

We assessed the linearity of the 3D EPI B_1_^+^ mapping method against varying RF transmit reference voltage (which determines the amplitude of the RF pulses and therefore the scaling of the B_1_^+^ maps). An extra dataset was acquired with a reference voltage manually modified by 10% and the corresponding B_1_^+^ map was rescaled by the same amount. The difference between this rescaled B_1_^+^ map and those acquired using automated settings was taken as a measure of the non-linearity of the method against varying B_1_^+^ field amplitudes.

We investigated the impact of the dynamic range optimization by using two ranges of α values on the first subject (using off-resonance minimization): α decreased from 240° to 100° in steps of 10° (experiment 1) and α decreased from 310° to 100° in steps of 15° (experiment 2). TR was 500 ms (600 ms) for experiment 1 (experiment 2) and the acquisition time was 3 min 48 s (4 min 34 s) per B_1_^+^ map. The B_1_^+^ value at each voxel was calculated using the 4 (3) pairs of SE/STE images (out of 15 pairs) yielding the maximum signal in the SE image.

The range of α values leading to the most robust B_1_^+^ maps was used in all later experiments on 2 subjects where we investigated the impact of off-resonance minimization using two RF pulse implementations. For one implementation, the duration of the RF pulses was set to 8680 µs for all nominal flip angle values α. For this implementation, the amplitude of the RF pulses was therefore proportional to the current α value. For the second implementation (*off-resonance minimization*), the duration τ of the RF pulse was set according to: 
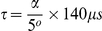
, yielding an RF voltage value close to the maximum allowed by the RF power amplifier. As a result, the same RF amplitude was used for all α values and the RF pulse duration was minimized. That is the RF amplitude was larger (and the RF duration was shorter) than for the first implementation for all but the maximum α value.

In summary, the optimal 3D EPI protocol (large range of nominal flip angles with off-resonance minimization) was used on a group of 3 subjects on which the linearity, reproducibility and accuracy (compared to the AFI method) of the method were assessed. The different aspects of the method optimization (flip angle range and off-resonance minimization) were tested on separate fractions of this group.

#### Phantom acquisitions

The larger range of nominal flip angle values (α decreased from 310° to 100° in steps of 15°) was also tested on an oil phantom. The duration τ of the RF pulse was set according to: 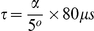 in order to minimize off-resonance effects. Parallel imaging was not implemented for this acquisition. As a result, the echo time TE was 50.98 ms, the mixing time TM was 47.13 ms and the total acquisition time was 7 min12 s. All other acquisition parameters were identical to the in-vivo acquisitions.

#### Bloch simulation of B_1_^+^ bias due to off-resonance

Numerical simulations of the Bloch equations were used to model the bias in measured B_1_^+^ due to spin off-resonance precession during the application of the RF pulses used by the 3D EPI method. The B_1_^+^ bias was simulated when off-resonance minimization was on or off using RF characteristics (shape, amplitude, duration and nominal value) identical to those used experimentally. The off-resonance bias was simulated for local B_1_^+^ values ranging from 40 p.u. to 150 p.u. by steps of 10 p.u. and B_0_ inhomogeneities ranging from 0 Hz to 200 Hz by steps of 10 Hz.

### AFI method

The following parameters were used for data acquisition with the AFI method: matrix size 64×60×48, image resolution 4×4×4 mm^3^, image orientation: (phase, read, partition) = (A-P, H-F, R-L), FOV 256×240×192 mm^3^, TE = 2.93 ms. The RF excitation pulse was a non-selective Hamming-filtered sinc pulse with a nominal time-bandwidth product of 1. The RF duration was 820 µs and the nominal flip angle value was 60°. The MR parameters for spoiling of transverse coherences were set following the recommendations given in [25]. The spoiler duration was set to 11 and 55 ms for TR_1_ and TR_2_ respectively. The spoiler amplitude was set to 26 mT/m, leading to gradient moment values A_G1_/A_G2_ = 286/1430 mT.ms/m [25]. The repetition times were set to the minimum achievable value given the spoiler duration i.e. 20 ms and 100 ms for TR_1_ and TR_2_ respectively. RF spoiling was used with a linear phase increment 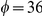
°. Partial Fourier (factor 6/8) was used along the partition direction. The total acquisition time was 4 min 32 s. Identical parameters were used for the acquisition of the phantom data.

### Reference 2D DAM method

A reference B_1_^+^ map was acquired on an oil phantom using the 2D DAM method described in [16]. The acquisition parameters were as follows: matrix size: 64×48, image resolution: 4 mm^3^, TE = 25 ms, image orientation (read, phase, slice) = (R-L, A-P, H-F). 48 slices were acquired and the repetition time was set to 25 s in order to avoid bias due to longitudinal relaxation. The presaturation pulse was a rectangular pulse of duration 500 us and its nominal flip angle value was set to 22° and 66°. The excitation RF pulse was a slice-selective sinc pulse of duration 2560 us. The total acquisition time was 40 min. Due to this long acquisition time this method was only used on the phantom.

## Results

**Figure 1:**
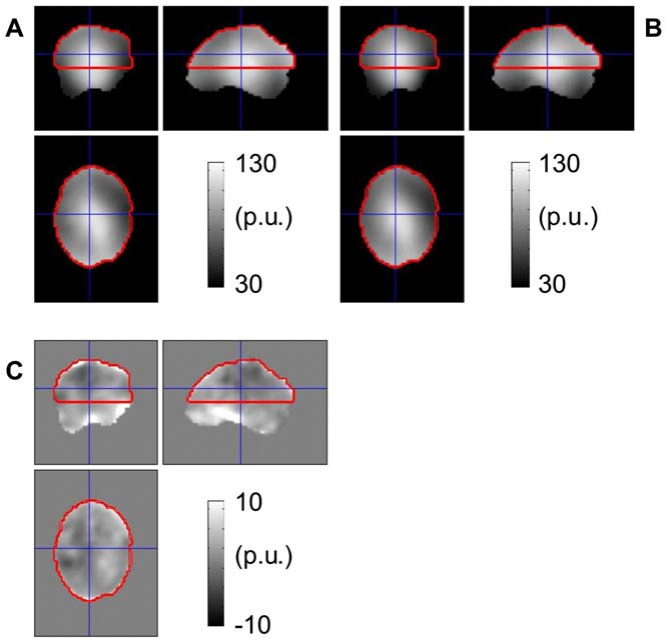
Comparison of 3D EPI and AFI methods. B_1_^+^ maps acquired with the 3D EPI (a) and AFI (b) methods. Difference between the B_1_^+^ maps acquired using the two methods (c). The red contour lines represent the superior part of the brain, used as a region of interest for quantitative comparison of both methods. Note that due to imperfect spoiling, the AFI method is expected to underperform in regions with low B_1_^+^ amplitudes such as the temporal lobes.

In the oil phantom data, the B_1_^+^ values obtained with the reference 2D DAM method were found to vary between 45 p.u. and 100 p.u.. The deviation between the reference 2D DAM method and 3D AFI method was −5.5±3 p.u. (mean and standard deviation) and the deviation between the reference 2D DAM method and 3D EPI method was −4.3±0.9 p.u. (mean and standard deviation). Figures 1a) and b) show B_1_^+^ maps acquired with the 3D EPI and AFI methods respectively, calculated as a percentage of the nominal flip angle (percent units = p.u.) to allow direct comparison between the B_1_^+^ maps. Figure 1c) represents the difference between the B_1_^+^ maps acquired using the 3D EPI and AFI methods. In the superior half of the field of view (represented as red contour lines in figure 1), the mean (standard deviation, ±SD) difference between the B_1_^+^ maps was 2.6 (±4.1) p.u. averaged across 3 subjects. In the inferior half of the field of view, larger differences were observed in the orbitofrontal cortex (OFC) and temporal lobes as expected from the limited dynamic range of the AFI method implementation [25].

**Figure 2:**
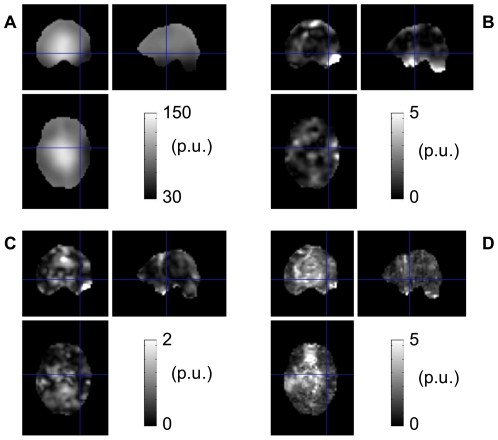
Precision and linearity of the 3D EPI method. Typical B_1_^+^ (a), non-linearity (b) instability (c) and SD_B1+_ (d) maps obtained with the optimal configuration using a maximum RF nominal value of 310° and off-resonance minimization.

Figure 2a) represents a typical whole brain B_1_^+^ map acquired with the larger flip angle dynamic range (100°–310°) and off-resonance minimization. The local B_1_^+^ values ranged from 40 to 150 p.u.. Figure 2b) and 2c) show typical non-linearity and instability maps. The brain-averaged non-linearity and instability of the B_1_^+^ maps were 1.6 and 0.7 p.u. averaged over all 3 subjects. The non-linearity of the local B_1_^+^ values was below 5 p.u. for 97.6% of the voxels inside the brain. The remaining 2.4% voxels had a mean non-linearity of 7.9 p.u. and were mostly located near the cerebellum and temporal lobes. The instability of the B_1_^+^ values was below 5 p.u. for 99.91% of the voxels. The remaining 0.09% voxels had a mean instability of 6.3 p.u.. Figure 2d) shows a typical SD_B1+_ map, representing the errors in the local B_1_^+^ values calculated using 3 SE/STE pairs at each voxel. SD_B1+_ values between 1 and 2 p.u. were found over most brain regions. Local regions with higher errors (∼5 p.u.) were found around the OFC due to residual off-resonance effects and temporal lobes.

**Figure 3:**
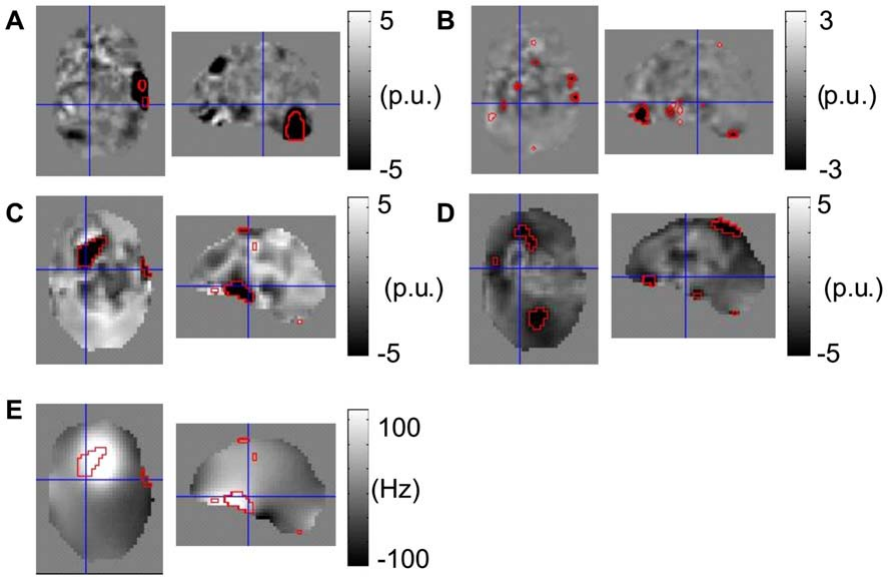
Improvements due to increase of the dynamic range of the method and off-resonance minimization. Changes in non-linearity (a) and instability (b) of the B_1_^+^ maps following an increase of the maximum RF nominal value to 310°. Change in non-linearity (c) and instability (d) of the B_1_^+^ maps after off-resonance minimization. Regions showing significant changes in non-linearity and instability are marked by red contour lines. B_0_ map acquired on the subject shown in c and d (e). The red contour lines in figure e show that off-resonance minimization improved the linearity of the method in areas with high B_0_ offsets.

Figures 3a) and 3b) represent changes in non-linearity and instability of the B_1_^+^ maps when the flip angle range was increased from 100°–240° to 100°–310°. The mean (±SD) reduction in non-linearity/instability was 3.1(±10.9)/0.1(±0.7) p.u. averaged over the brain. 3.3/4.6% of voxels showed changes above a significance threshold of mean–2xSD and the mean reduction was 53/2.4 p.u. for these voxels. Figures 3c) and 3d) represent changes in non-linearity and instability of the B_1_^+^ maps when off-resonance minimization was used. The changes in non-linearity/instability were 0.2(±1.7)/−0.9(±0.8) p.u. averaged over 2 subjects. 4.1/5% of the voxels showed changes above the significance threshold and the mean reduction was 5.2/3.1 p.u. for these voxels. Regions showing significant changes in non-linearity and instability are marked by red contour lines in figure 3. The most significant improvements due to the increase of the flip angle range were found in the temporal lobes and cerebellum, where the local B_1_^+^ values were lowest. The most significant improvements following off-resonance minimization were found in the OFC, where B_0_ gradients and off-resonance effects were highest due to susceptibility effects (see figure 3e). The Bloch equation simulations of off-resonance spin precession during the application of the RF pulses showed a bias in the calculated B_1_^+^ value above 5 p.u. for B_0_ inhomogeneities >∼140 Hz and 60p.u.≤B_1_^+^≤90p.u. without off-resonance minimization. The off-resonance bias was found below 5p.u. for B_1_^+^<60p.u. because large local flip angles were not achieved for these B_1_^+^ values given the range of nominal flip angle values used in-vivo. The main effect of off-resonance minimization was to reduce the off-resonance bias by ∼7 p.u. in regions where B_1_^+^∼80p.u. and B_0_ inhomogeneities >∼150 Hz. This is in good agreement with the experimental data showing a reduction in off-resonance bias around the OFC when off-resonance minimization is used. Results from the Bloch simulations showed that a bias of up to ∼8p.u. might remain in the B_1_^+^ maps when off-resonance minimization is used where B_0_ inhomogeneities reach ∼200 Hz.

## Discussion

We have presented a 3D EPI method for optimized whole brain B_1_^+^ mapping at 7T. To our knowledge, this study is the first systematic analysis of the accuracy and reproducibility of a whole-brain B_1_^+^ mapping method at 7T (as determined by a literature search of the PubMed database for titles and abstracts containing the terms “7T AND (B1 OR RF)”). The accuracy of the 3D EPI method was assessed on a phantom by comparison with a reference technique. This comparison was not performed in-vivo due to the lack of a fast reference method comprehensively validated at 7T. The 3D EPI method was validated in-vivo using three complementary criteria: reproducibility across repetitions, accuracy (measured as the deviation from the well-established 3D AFI method [12], [16]) and linearity against varying reference voltage. Improvements to the existing 3D EPI method [16] were introduced in order to address the challenges of B_1_^+^ mapping at 7T. The use of RF pulses with large amplitudes reduced bias due to off-resonance spin precession in brain regions with strong B_0_ inhomogeneities (*off-resonance minimization*). The large range of RF nominal values increased the dynamic range of the method, enabling accurate B_1_^+^ mapping over the large range of B_1_^+^ values present in the brain at 7T. The total acquisition time was minimized by extensive use of parallel imaging (4 min34 s+2 min B_0_ map).

### Validation of the 3D EPI method

The 3D EPI and 3D AFI methods were compared with a reference 2D DAM method on an oil phantom. A deviation of −4.3±0.9 p.u was observed between the 3D EPI and the 2D DAM method and a deviation of −5.5±3 p.u. was found between the 3D AFI and the 2D DAM method. Note that parallel imaging was not implemented for the 3D EPI acquisition on the oil phantom, degrading the quality of the results obtained for this method. In-vivo quantitative measures of the accuracy, reproducibility and linearity of the optimized 3D EPI method were extracted from a group of 3 subjects.

Since no fast reference technique has been validated in-vivo at 7T, accuracy estimates were obtained by calculating the deviations between the 3D EPI and 3D AFI methods. The acquisition parameters of the 3D AFI method were carefully set in order to achieve maximal spoiling of the transverse coherences and highly accurate B_1_^+^ maps [25]. Only small (2.6±4.1 p.u.) differences in B_1_^+^ values were observed between the two methods in the superior part of the brain. B_1_^+^ values larger by 5 to 10 p.u. were measured with the 3D EPI method in the temporal lobes, where the local B_1_^+^ values were particularly low. This is in agreement with systematic underestimation of the B_1_^+^ field by the 3D AFI method due to suboptimal spoiling conditions where the actual flip angle is low [25] and supports the claim that our method maps the B_1_^+^ field accurately over the entire brain. The discrepancies between the two methods in the OFC most likely stem from the off-resonance sensitivity of both methods (see Results section for estimates of the sensitivity of the 3D EPI method to off-resonance effects obtained from numerical Bloch Equation simulations).

The linearity of the 3D EPI B_1_^+^ mapping method against varying RF transmit reference voltage is a necessary though not sufficient condition to demonstrate the robustness of the method and is therefore complementary to the reproducibility and accuracy measures supplied here. An average non-linearity of 1.6 p.u. was found across the 3 scanned subjects, demonstrating a highly linear relationship between the measured B_1_^+^ maps and RF transmit voltage. The 10% increase in reference voltage used for the linearity test proved to be sufficient in order to demonstrate the improvements from our optimization procedure and was significantly higher than the level of inaccuracy (compared with the 3D AFI method), reproducibility and non-linearity demonstrated here. If a stronger variation of the reference voltage is desired to enhance the sensitivity of the test, special attention should be given to the maximum RF voltage in order to avoid clipping of RF pulses.

A measure of the reproducibility of the 3D EPI method was obtained by calculating the voxel-wise standard deviation of the B_1_^+^ maps over three successive acquisitions. An average level of instability of 0.7 p.u. was observed, illustrating the robustness of the method against physiological effects.

### Optimization of the 3D EPI method

A large number of α values was used to allow dense sampling of the large B_1_^+^ inhomogeneities present at 7T and increase the dynamic range of the technique. A sub-sample of SE/STE images was selected for each voxel to avoid low signal to noise in the calculation of the B_1_^+^ values. This implied a reduction in data sampling efficiency but enabled accurate and precise B_1_^+^ mapping over the whole brain. An improved modelling and weighted fitting procedure may allow for use of the whole dataset and increase precision further. Highly accelerated image acquisitions helped keep the total acquisition time sufficiently short (4 min34 s+2 min B_0_ map) so that B_1_^+^ mapping can remain only a small fraction of a larger scanning protocol. The increased dynamic range of the optimized method led to a 53 p.u. reduction in the non-linearities of the 3D EPI method against RF transmit voltage in the temporal lobes and cerebellum and is essential for whole-brain B_1_^+^ mapping at 7T.

Minimization of off-resonance effects consisted of the use of RF pulses with maximal amplitude and minimal duration for all nominal values. This led to a reduction of the non-linearities by 5.2 p.u. in the OFC, in good agreement with the results from the Bloch Equation simulations. Further improvements to the method might involve correcting for the effect of off-resonance precession on the local B_1_^+^ values using the results of the Bloch simulations presented here and the acquired B_0_ maps.

### Considerations

The average non-linearity and reproducibility of the B_1_^+^ maps after optimization was 1.6 p.u. and 0.7 p.u. averaged over the whole brain and across subjects. However, local regions with non-linearities greater than 5 p.u. remained in the temporal lobes and cerebellum. A larger maximum α value (leaving the spacing between consecutive values unchanged) would improve the robustness of the method in these regions without compromising the quality of the B_1_^+^ maps in other brain regions. Alternatively, other methods may be suitable for fast and robust whole brain B_1_^+^ mapping at 7T (e.g. [12], [14], [16], [26]), although a systematic analysis of the performance of these methods at UHF has not been reported. The 2D STEAM method described in [16], [26] is particularly fast but sensitive to physiological artefacts and requires independent calibration. The 3D STEAM method presented in [15] might alleviate these difficulties and benefit from the shorter acquisition time. However, the problems related to off-resonance and large B_1_^+^ deviations present at 7T might also prove problematic for this technique. Long TR gradient-echo techniques such as in [17] yield robust B_1_^+^ estimates due to their simplicity. However, their long acquisition time (∼20 mins per slice, see e.g. [17]) renders these methods impractical for in-vivo applications.

The optimized B_1_^+^ mapping protocol described here could be implemented on all subjects without clipping of the RF pulses and/or exceeding the SAR safety limits. The reference RF voltage was typically ∼315 V for the subjects scanned, leading to a maximum RF voltage of ∼450 V. This was sufficiently low to accommodate a 10% increase in reference voltage to test the linearity of the method without clipping the RF pulses or exceeding the SAR limit. A maximum nominal flip angle of 340° should therefore be achievable. However, if higher nominal flip angle values are desirable, longer RF pulses should be used with longer TR values in order to comply with safety limitations. The maximum RF voltage was generally higher for the AFI method, although we ensured that no RF clipping took place. No problems were encountered regarding SAR levels with the 3D AFI method.

Note that the 3D EPI method can easily be tuned to produce high quality B_1_^+^ maps in one specific region of interest only by setting the range of nominal flip angles appropriately. The number of required nominal flip angles might be significantly reduced as a result, leading to a significant reduction in acquisition time. Although this method has not been tested outside the brain, the possibility of tuning the nominal flip angle values according to a specific region of interest might prove advantageous when other body parts are targeted. However, specific features of the body part of interest (size, tissue density, B_0_ homogeneity, …) should be considered with care.

Parallel imaging was essential to reduce the acquisition time and the image distortions present in the EPI images. Despite parallel imaging, image distortions remained visible in the SE and STE images which required correction. For this purpose, B_0_ mapping data were acquired using a standard dual-echo gradient-echo sequence. The two corresponding echo times were carefully chosen to provide a high SNR, result in a fat signal in-phase across both echo images and avoid phase wrapping problems in the B_0_ map calculated as part of the unwarping procedure. Due to the lack of a distortion correction software on the scanner console, image distortion was implemented offline [16], [23].

### Conclusion

We have presented an optimized SE/STE 3D EPI method for mapping of the RF transmit field B_1_^+^ at 7T. A robust offline unwarping procedure was used in order to correct for image distortions. The dynamic range of the method was increased to match the higher level of B_1_^+^ inhomogeneities at 7T and off-resonance effects were minimized using RF pulses with large amplitudes and short durations. The improvements induced by our optimization were most significant in the temporal lobes, cerebellum and orbitofrontal regions, which are notoriously problematic brain regions at ultra high fields. Whole brain B_1_^+^ maps were obtained in a total acquisition time of 4 min34 s (+2 min B_0_ map) with non-linearity and reproducibility of 1.6 p.u. and 0.7 p.u. respectively and deviations of 2.6 p.u. from the 3D AFI method in brain regions where the latter method performed satisfactorily.
